# Synergistic impact of mutations in Hepatitis B Virus genome contribute to its occult phenotype in chronic Hepatitis C Virus carriers

**DOI:** 10.1038/s41598-017-09965-w

**Published:** 2017-08-29

**Authors:** Rajiv Kumar Mondal, Mousumi Khatun, Priyanka Banerjee, Alip Ghosh, Sumanta Sarkar, Amal Santra, Kausik Das, Abhijit Chowdhury, Soma Banerjee, Simanti Datta

**Affiliations:** 1 Centre for Liver Research, School of Digestive and Liver Diseases, Institute of Post Graduate Medical Education and Research, Kolkata, India; 2 Department of Hepatology, School of Digestive and Liver Diseases, Institute of Post Graduate Medical Education and Research, Kolkata, India

## Abstract

We characterized occult HBV (OHBV) from hepatitis B surface antigen (HBsAg)-negative chronic HCV carriers of Eastern India to explore the impact of genomic variability of HBV in causing undetectability of HBsAg and low viremia that define the occult phenomenon. Screening of sera samples revealed the presence of OHBV in 17.8% of HCV-infected patients. Determination of full-length OHBV sequences and comparison with that from HBsAg-positive carriers led to the detection of distinct substitutions/mutations in PreS2, S, P and X ORFs and in X-promoter and Enhancer-II of OHBV. These mutations were introduced in wild-type HBV and their effects were evaluated by transfection in Huh7 cells. *In vitro* assays demonstrated that S-substitutions resulted in antigenically modified HBsAg that escaped detection by immunoassays whereas those in ORF-P caused significant decline in viral replication. Impairment in Enhancer-II and X-promoter activities were noted due to occult-associated mutations that generated reduced pregenomic RNA and intracellular HBV-DNA. Additionally, Enhancer-II mutations altered the small to large surface protein ratio and diminished extracellular HBV-DNA and HBsAg secretion. Further, mutations in PreS2, X and enhancer-II increased Grp78-promoter activity, suggesting that OHBV could trigger endoplasmic reticulum stress. Thus viral mutations contribute synergistically towards the genesis of occult phenotype and disease progression.

## Introduction

Infection by Hepatitis B virus (HBV) constitutes the most common cause of chronic liver disease including chronic hepatitis, cirrhosis and hepatocellular carcinoma (HCC)^1^. The current routine diagnosis of HBV infection relies on the detection of Hepatitis B surface antigen (HBsAg) in the patients’ sera and its disappearance has been correlated with clearance of viremia and resolution of liver injury^1^. However, with the availability of highly sensitive genome amplification methods, low-level of HBV-DNA could be detected in sera or liver tissues of HBsAg-negative patients and this particular form of viral persistence has been commonly termed as “occult” HBV infection (OBI)^2^. The molecular mechanisms underlying the occult phenomenon still remain ambiguous. Both viral and host factors have been implicated in the induction of OBI, although recent studies have placed more emphasis on the role of host immunological and epigenetic factors in this regard^3–5^.

OHBV had been identified in patients with chronic hepatitis C virus (HCV) infection, given that these two viruses share the same modes of transmission. It had been suggested that OHBV may interfere with the clinical outcome of chronic hepatitis C and favor or accelerate the evolution to cirrhosis or development of HCC^2, 3^. In addition the presence of OHBV had been correlated with poor response of HCV patients to interferon therapy^3^ and their propensity for reactivation could undermine the success of liver transplantation^6^ that is often recommended for patients with HCV-related cirrhosis. The recent introduction of a variety of direct-acting antiviral agents has made HCV cure possible but has increased the likelihood of occult HBV reactivation in co-infected patients that may even lead to fatal hepatitis B^7, 8^. Hence identifying HCV carriers carrying OHBV and expansion of the knowledge about OHBV biology are needed to guide treatment decisions, to design improved methods of OHBV detection and to fathom the pathogenic potential or the clinical implications of the occult virus. The available data on the frequency of OBI in HCV patients are widely divergent^9, 10^ and there has been no study on the molecular characterization of full-length OHBV in HCV carriers. India has over 40 million HBV carriers^11^ and about 15 million HCV carriers^12^ and the incidence of HBV and HCV co-infection in chronic liver disease population had been variously reported between 3–56%^13^. But there is limited information on the prevalence and genetic features of OHBV in chronically HCV-infected Indian patients. Hence, the present study sought to determine the OBI prevalence in chronic HCV carriers of Eastern India and to carry out comprehensive molecular and functional analyses of OHBV to explore the possible role of genomic variability in causing (1) absence of HBsAg and (2) low viremia, the two defining features of OBI. In addition, the contribution of OHBV towards disease pathogenesis was also investigated.

## Results

### Prevalence of serological markers of HBV among HCV patients

A total of 213 treatment-naïve, HBsAg-negative patients with chronic HCV infection were recruited in the study and their demographic and clinical features are summarized in Table 1. The prevalence of antibody to hepatitis B core antigen (anti-HBc) and antibody to HBsAg (anti-HBs) were tested in all HCV carriers. It was observed that ~15% of these patients were positive for both anti-HBs and anti-HBc, ~24% had isolated anti-HBc, ~11% showed only anti-HBs sero-positivity, while ~51% of the patients did not possess any anti-HBV serological marker (Table 1).

**Table 1:** Clinical, demographic and biochemical data of HCV patients with or without OBI.

	Total HCV Patients	HCV patients with OBI	HCV patients without OBI	p value
	n = 213	n = 38	n = 175	
Age (Year), median (range)	52 (6–80)	55 (14–78)	50 (6–80)	>0.05^a^
anti-HBs+/anti-HBc+ (%)	31 (14.6)	8 (21.1)	23 (13.1)	>0.05^b^
anti-HBs−/anti-HBc+ (%)	51 (23.9)	7 (18.4)	44 (25.1)	>0.05^b^
anti-HBs+/anti-HBc− (%)	23 (10.8)	5 (13.2)	18 (10.3)	>0.05^b^
anti-HBs−/anti-HBc− (%)	108 (50.7)	18 (47.4)	90 (51.4)	>0.05^b^
Sex, M:F	144:69	28:10	116:59	>0.05^b^
ALT (IU/L), median (range)	57.5 (11–237)	57 (19–117)	58 (11–237)	>0.05^a^
AST (IU/L), median (range)	76 (19–195)	76.65 (22–111)	76 (19–195)	>0.05^a^
Total bilirubin (mg/dL), median (range)	0.97 (0.4–5.8)	1.14 (0.5–5.4)	0.9 (0.4–5.8)	>0.05^a^
Alkaline phosphatase (IU/L), median (range)	141 (28–418)	140 (47.1–400)	151 (28–418)	>0.05^a^
OHBV-DNA (copies/mL), median (range)	NA	237.1 (10–1056)	NA	NA
HCV-RNA (copies/mL), median (range)	92867 (100–5 × 10^6^)	49100 (100–6.5 × 10^5^)	281050 (100–5 × 10^6^)	>0.05^a^

OBI, Occult HBV infection; ALT, alanine aminotransferase; AST, aspartate aminotransferase; IU, international unit; NA, Not Applicable.

^a^Mann Whitney test, p value.

^b^Fisher’s exact test, p value.

### Detection of OBI

By applying the currently accepted criteria for the identification of OBI, which entails the positive detection of HBV-DNA in at least two out of at least three different genomic regions of HBV analyzed by nested PCR^2^, 38 (17.8%) out of 213 HCV patients were found to carry OHBV. Further, among these 38 OBI-positive patients, the presence of both anti-HBs and anti-HBc were noted in 8 (~21%) patients; isolated anti-HBc was identified in 7 (18%), anti-HBs alone in 5 (13%) while 18 (47%) patients had no evidence of prior HBV exposure or remote infection (anti-HBs−/anti-HBc−) (Table 1). The median HBV DNA level in these 38 HCV patients was 237 copies/ml (range, 10–1056 copies/ml) (Table 1).

### OHBV genotype

Amplification and full-genome sequencing of OHBV was attempted from 38 OBI-positive samples of HCV-infected patients. However complete sequences were obtained from 19 OHBV isolates. Phylogenetic analysis revealed that 17 (89.5%) out of 19 OHBV isolates clustered with genotype D, whereas the remaining two were of genotype C (Supplementary Figure S1). Subsequent analyses of genetic diversification and properties of OHBV were carried out exclusively on 17 OHBV/D isolates.

### Identification of Occult-associated mutations in HBV

To identify distinct nucleotide/amino acid changes that characterize OHBV, the 17 full-length OHBV/D sequences determined in the study were compared with 105 HBV/D sequences derived from HBsAg-positive chronic HBV carriers in different parts of the world (WRLD) and 62 HBV/D sequences from Eastern India (EI) and the following mutations were observed (Table 2).

**Table 2:** Frequencies of mutations across the complete genome of OHBV/D as compared to HBV/D from HBsAg-positive carriers.

Site in HBV Amino Acid/Nucleotide Substitution	OHBV/D sequences determined in this study (n = 17)(%)	HBV/D sequences from HBsAg-Positive carriers of Eastern India (EI) (n = 62)(%)	HBV/D sequences from HBsAg-Positive carriers of other geographical regions (WRLD) (n = 105)(%)	p2 (OHBV vs HBV/D_EI)^a^	p1 (OHBV vs HBV/D_WRLD)^a^
**PreS1/PreS2/S-ORF**					
PreS2-P36L	11(64.9)	10(16)	1(0.9)	1.97E-04	1.01E-10
PreS2-I42L	14(82.6)	16(25.6)	27(25.6)	3.59E-05	1.25E-05
P36L+I42L	11(64.9)	9(14.4)	1(0.9)	1.03E-04	1.01E-10
sT125M	16(94.4)	22(35.2)	20(19)	1.37E-05	2.48E-09
sP127T	17(100)	43(68.8)	39(37)	8.29E-03	3.82E-07
sT125M+sP127T	16(94.4)	21(33.6)	20(19)	1.02E-05	2.48E-09
**X-ORF**					
R26C	11(64.9)	11(17.6)	19(18)	3.57E-04	1.70E-04
P33S	11(64.9)	19(30.4)	34(32.3)	2.19E-02	1.46E-02
R26C+P33S	11(64.9)	11(17.6)	10(9.5)	3.57E-04	1.84E-06
**ORF-P**					
spE1D	12(70.8)	10(16.1)	13(12.4)	3.65E-05	1.38E-06
spI59F	10(59)	13(20.9)	22(20.9)	5.17E-03	2.23E-03
spL80F	11(64.9)	14(22.5)	35(33.3)	2.26E-03	1.68E-02
spL116F	10(59)	10(16.1)	13(12.3)	8.92E-04	7.26E-05
rtA21S	11(64.9)	15(24.2)	26(24.7)	3.01E-03	2.97E-03
rtF122L	10(59)	14(22.5)	24(22.8)	6.87E-03	6.33E-03
rtH124N	13(76.7)	9(14.5)	5(4.7)	2.58E-06	1.56E-10
rtQ130P	10(59)	17(27.4)	22(20.9)	2.19E-02	2.23E-03
rtN131D	10(59)	9(14.5)	4(3.8)	4.97E-04	1.11E-07
rtD263E	14(82.6)	28(45.1)	40(38)	1.20E-02	1.04E-03
rtV278I	11(64.9)	8(12.9)	14(13.3)	5.10E-05	4.57E-05
rhL30S	11(64.9)	7(11.3)	10(9.5)	2.35E-05	1.84E-06
rhL55I	14(82.6)	27(43.5)	38(36.1)	5.79E-03	4.53E-04
**X-Promoter**					
T1050G	12(70.6)	12(19.4)	12(11.4)	1.29E-04	7.52E-07
A1053G	11(64.7)	11(17.7)	10(9.5)	3.57E-04	1.84E-06
C1059T	11(64.7)	13(21)	14(13.3)	1.89E-03	1.85E-05
C1350A	11(64.7)	8(12.9)	2(1.9)	5.10E-05	6.21E-10
**Enh-II**					
C1637A	11(64.7)	9(14.5)	9(8.6)	1.03E-04	9.23E-07
T1676A	12(70.6)	13(21)	7(6.7)	2.28E-04	1.77E-08

^a^Fisher’s exact test, p value; ORF, open reading frame; sp, spacer; rt, reverse transcriptase; rh, RNaseH.

#### ORF-PreS1/PreS2/S

The HBV surface gene consists of a single ORF divided into three coding regions: preS1, preS2 and S. A combination of all three regions form the Large surface protein (LHBs), the PreS2 and S domains form the middle (MHBs) while the S domain codes for the small surface protein (SHBs or HBsAg). The amino acid sequences of the surface proteins of OHBV/D revealed that the substitutions, P36L and I42L in PreS2 occurred at significantly high frequency, either singly or dually, relative to that from HBsAg-positive carriers (Table 2). In addition, two substitutions, T125M and P127T within the major B-cell epitope cluster [amino acid (aa) 124–147] (referred to as “a”-determinant)^4, 5^ of S-ORF had significantly higher prevalence in OHBV/D than in overt HBV, irrespective of their geographical origin (Table 2).

#### ORF-X

Within ORF-X, two substitutions, R26C and P33S were perceived at significantly higher rates (64.9%) in OHBV/D in comparison to HBV from HBsAg-positive individuals (Table 2). Of these, P33S lies in B-cell epitope (aa.29–48) while R26C falls in non-epitope region of X.

#### ORF-P

A total of 13 substitutions in ORF-P, including four in spacer (sp), seven in rt and two in rh domains were detected at a significantly high proportion in OHBV isolates as compared to that from HBsAg-positive cases (Table 2). As the PreS/S-ORF completely overlaps ORF-P, two substitutions, I42L in PreS2 and P127T in S corresponded to two additional changes, spH153L and rtS135Y respectively in ORF-P and had distribution patterns identical to those of PreS1 and S variants in the study groups.

#### HBV Regulatory Regions

Analysis of the sequences of HBV regulatory regions, namely, basal core promoter (BCP; nt.1742–1849), PreS1-promoter (nt.2704–2823), PreS2/S-promoter (nt.2978–3207), Enh-I (nt.1074–1234) and negative regulatory element (nt.1613–1636) of OHBV showed no significant difference from that derived from HBsAg-positive cases. However within X-promoter (nt.957–1354), four mutations, T1050G, A1053G, C1059T and C1350A were found to be significantly high in OHBV as compared to that from HBsAg-positive (or overt) infections in same or different geographical regions (Table 2). However, owing to the overlap between X promoter and ORF-P, one substitution in rt (rtV278I) and two substitutions in rh domain (rhL30S and rhL55I) of ORF-P (Table 2) resulted in four additional changes (G961A, C1249T, T1250C and C1324A) in X-promoter. In addition, two mutations, C1637A and T1676A were detected within the Enh-II (nt.1636–1741) that had significantly higher prevalence in OHBV than in reference sequences (Table 2).

#### Combination of occult-associated mutations

We evaluated the number of OHBV isolates that harboured all the above mutations synchronically. All identified mutations within coding and regulatory regions were found to co-exist in 10 (58.8%) out of 17 OHBV/D.

### Effect of S-substitutions on Antigenicity, expression and detection of HBsAg

**Figure 1:**
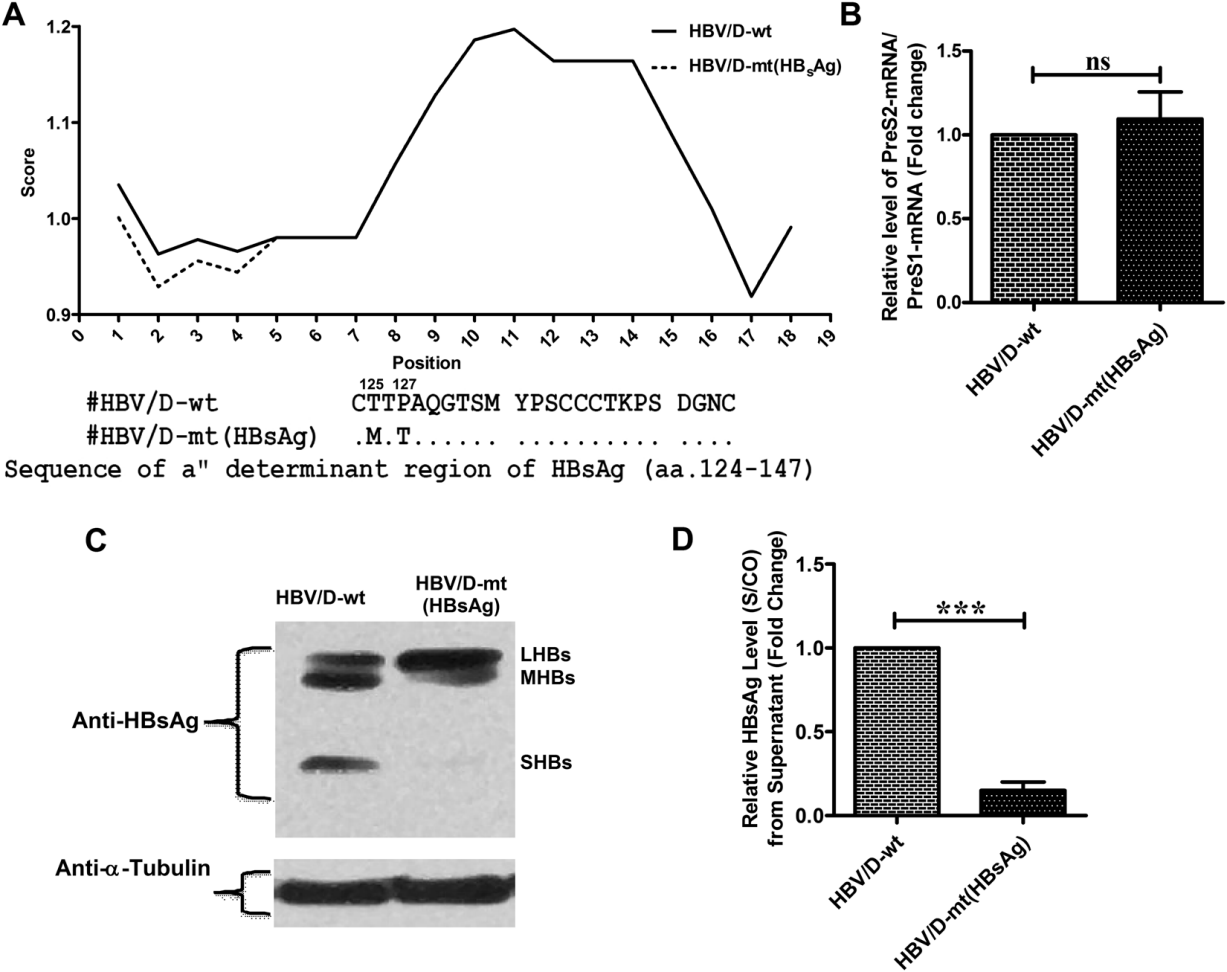
Effects of substitutions in ORF-S on HBsAg antigenicity and its detection. (**A**) Antigenicity plot of “a” determinant (aa.124–147) of HBsAg showing decreased antigenicity due to substitutions T125M and P127T as obtained by Kolaskar and Tongaonkar Antigenicity Prediction method. The relative ratio of PreS2/S and PreS1 mRNA determined by quantitative real-time PCR (**B**), the expression of HBV envelope proteins by immunoblot assay with mouse monoclonal anti-HBs primary antibody [the cropped gels are shown here for clarity while the full-length blots are presented in Supplementary Figure S2 (**A**); α-Tubulin served as the loading control] (**C**) and HBsAg level [Signal(S)/Cutoff(CO)] in culture supernatant estimated by ELISA (**D**), following transfection of wt-HBV (HBV/D-wt) and mutant construct [HBV/D-mt(HBsAg)], having T125M and P127T substitutions in Huh7 cells. Paired t-test p values; ***p < 0.0005, ns; non-significant.

We first examined the impact of the substitutions, T125M and P127T on the antigenicity of the B-cell epitope (aa 124–147) of HBsAg by Kolaskar and Tongaonkar Antigenicity Prediction method and observed that both substitutions resulted in decreased antigenicity relative to wild-type (wt) residues (Fig. 1A), suggesting that these changes may affect the binding of HBsAg to anti-HBs and its detection by immunoassays.

Further, introduction of these two S-mutations in wild-type full-length HBV and transfection of both the wild-type and mutant HBV genomes into Huh7 cells demonstrated that the presence of T125M and P127T in HBsAg did not alter the levels of PreS2/S transcripts relative to wt, as determined by Real time PCR (Fig. 1B) but resulted in significant reduction in HBsAg detection both intracellularly by immunoblotting (Fig. 1C) and in culture supernatant by ELISA (p < 0.0001) (Fig. 1D).

### Effect of substitutions in ORF-P on HBV replication

**Figure 2:**
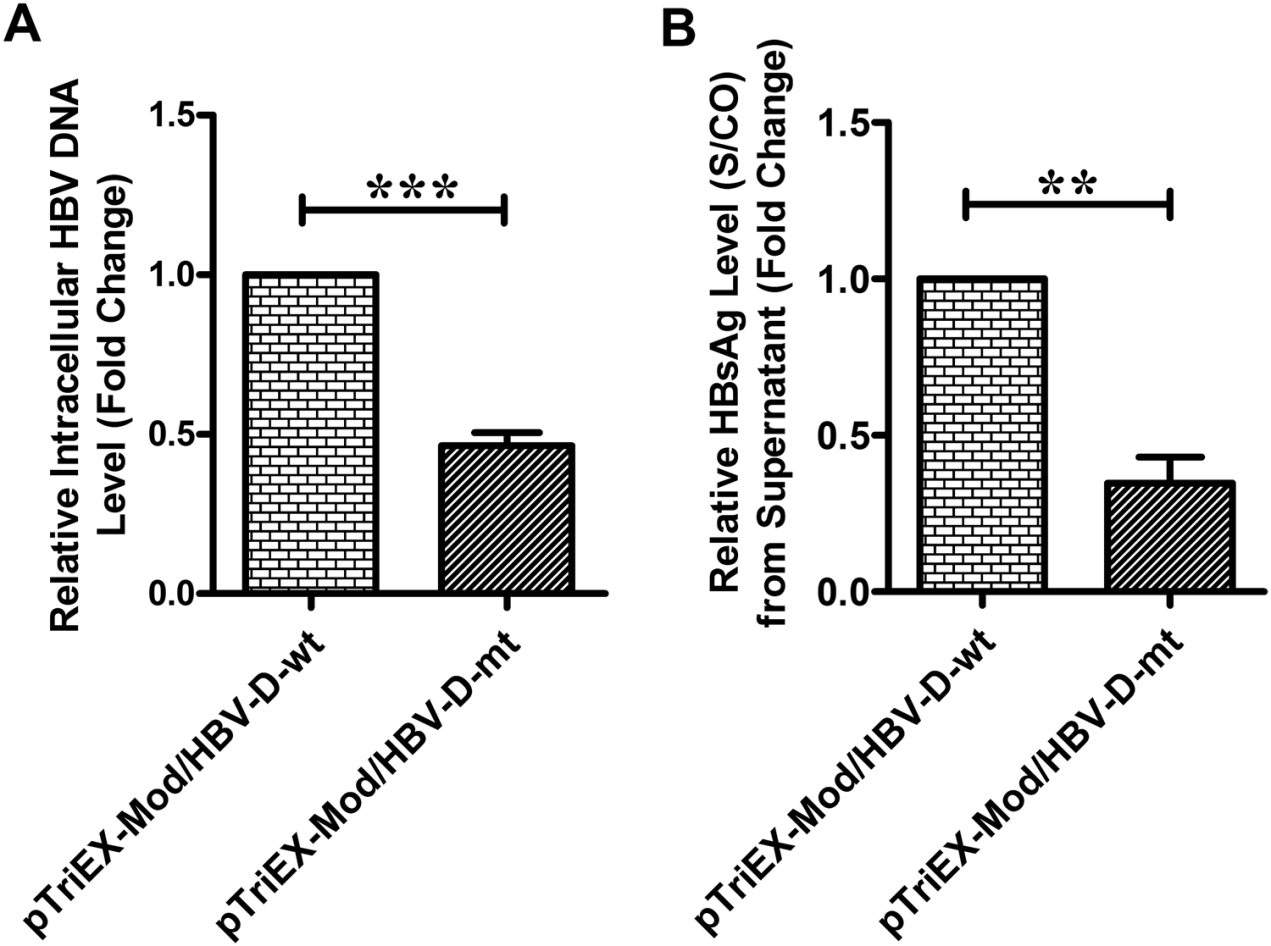
Effects of substitutions in reverse transcriptase (rt) and RNaseH (rh) domains of ORF-P on HBV replication. The relative intracellular HBV-DNA level measured by quantitative real-time PCR (**A**) and HBsAg level [Signal(S)/Cutoff(CO)] in culture supernatant estimated by ELISA (**B**), following transfection of the wt-HBV (pTriEx-Mod/HBV-D-wt) and mutant construct (pTriEx-Mod/HBV-D-mt), having rt and rh substitutions in Huh7 cells. Paired t-test p values; **p < 0.005, ***p < 0.0005.

To investigate whether the substitutions detected in ORF-P, particularly in the rt and rh domains of OHBV affect its replication capacity in comparison to the wild-type isolates lacking these substitutions, HBV constructs, pTriEXMod/HBV-D-wt and pTriEXMod/HBV-D-mt, differing only in rt and rh domains were generated, such that pTriEX-Mod/HBV-D-mt harboured seven substitutions in rt and two substitutions in rh domains, whereas pTriEXMod/HBV-D-wt carried wild-type residues at analogous positions. Transfection of these constructs into a Huh7 cell line and quantification of intracellular core-associated HBV DNA indicated that HBV expressed from pTriEX-Mod/HBV-D-mt had a mean ~2.15 fold lower replication efficiency than HBV from pTriEX-Mod/HBV-D-wt (p < 0.0001) (Fig. 2A). Consistent with these data, ~2.9-fold decrease in HBsAg (p = 0.0015) was noted in culture supernatant when pTriEX-Mod/HBV-D-mt was used (Fig. 2B).

### Effect of Enh-II mutations on HBV replication and HBsAg expression

**Figure 3:**
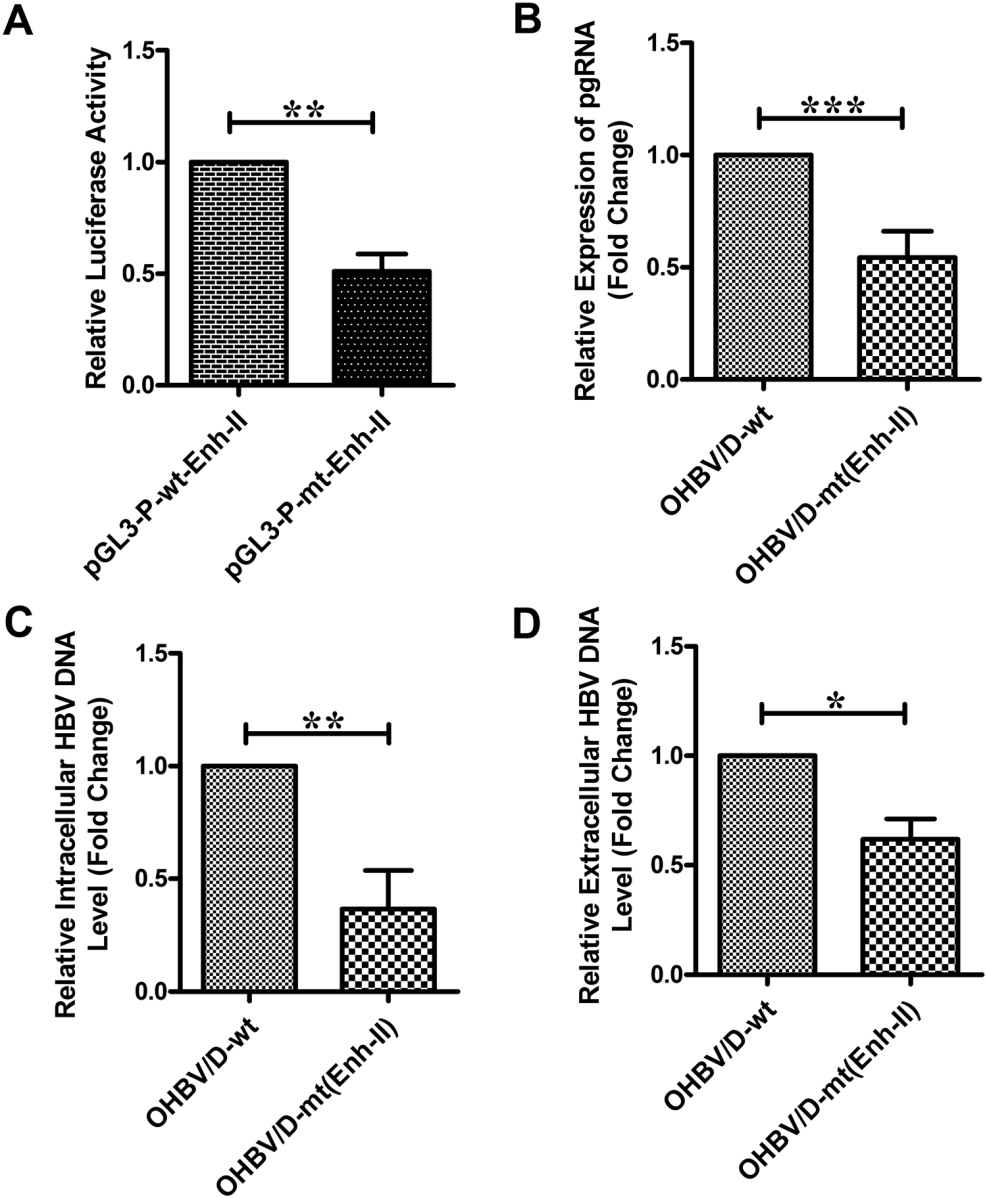
Effects of substitutions in Enh-II on enhancer activity, pgRNA expression and HBV-DNA level. (**A**) Relative luciferase activities of the constructs pGL3-P-wt-Enh-II and pGL3-P-mt-Enh-II carrying wild-type and mutant Enh-II elements respectively in pGL3-promoter vector. Relative expression of pgRNA (**B**), intracellular HBV-DNA level (**C**) and extracellular HBV-DNA level (**D**), as measured by quantitative real-time PCR, following transfection of the full-length, wild-type HBV/D (HBV/D-wt) and corresponding Enh-II-mutated HBV [HBV/D-mt(Enh-II)] in Huh7 cells. Paired t-test p values; *p < 0.05, **p < 0.005, ***p < 0.0005.

Enh-II is known to stimulate transcription from different HBV promoters, importantly BCP and PreS2/S-promoter and thereby regulates the expression of HBV genes and viral replication^14^. We first sought to determine the effects of the two mutations, C1637A and T1676A on the enhancer activity, for which the wild type and mutant Enh-II element was separately cloned in enhancer-less pGL3 promoter vector. It was noted that the level of luciferase activity directed by mutant-Enh-II was significantly less than that conferred by wild-type Enh-II (Fig. 3A) (p = 0.0011), suggestive of a decline in enhancer activity in OHBV.

We next explored whether the Enh-II mutations could impact the replication efficiency of HBV and we transfected the full-length wild-type HBV and its mutant counterpart (carrying the above two mutations) into Huh7 cells. As shown in Fig. 3B–D, Enh-II-mutated HBV demonstrated ~2 fold reduced levels of pregenomic RNA (pgRNA), transcribed from BCP and similar decline in intracellular and extracellular HBV-DNA as compared to wt-HBV (p < 0.05).

**Figure 4:**
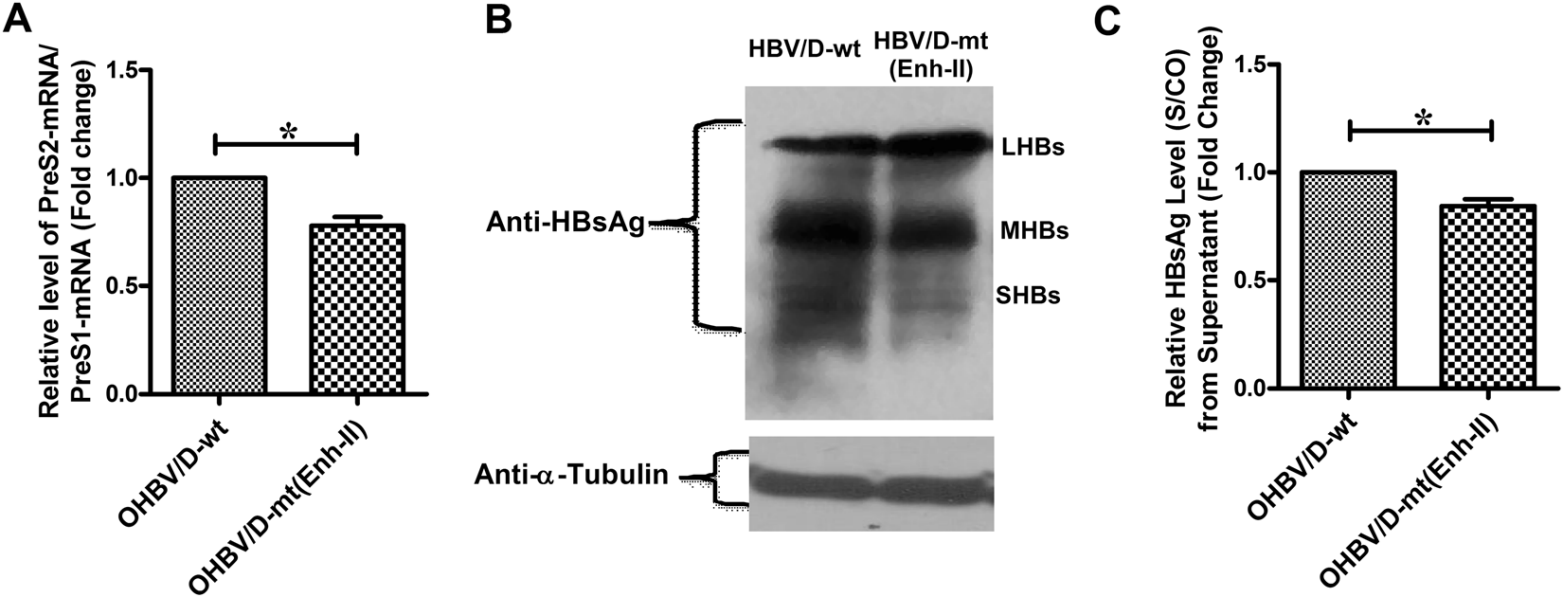
Effects of substitutions in Enh-II on the envelope protein expression. Relative ratio of PreS2 and PreS1-mRNA as determined by quantitative real-time PCR (**A**), expression of HBV envelope proteins by immunoblot assay with mouse monoclonal anti-HBs primary antibody [the cropped gels are shown here for clarity while the full-length blots are presented in Supplementary Figure S2 (**B**); α-Tubulin served as the loading control] (**B**) and HBsAg level [Signal(S)/Cutoff(CO)] in culture supernatant estimated by ELISA (**C**), following transfection of the full-length, wild-type HBV/D (HBV/D-wt) and corresponding Enh-II-mutated HBV [HBV/D-mt(Enh-II)] in Huh7 cells. Paired t-test p values; *p < 0.05.

We also evaluated the expression of HBV surface proteins in Huh7 cells transfected with wt- or Enh-II-mutated HBV to discern whether the Enh-II mutations alter the natural stoichiometry between the LHBs and SHBs that is critical for secretion of virions and HBsAg^15^. It was noted that the PreS2/S transcript as well as intracellular SHBs was reduced in presence of Enh-II mutations while the levels of PreS1-mRNA and LHBs were comparable to that of wt-HBV, thereby implying a low SHBs:LHBs ratio in Enh-II-mutated virus (Fig. 4A and B). Concurrently, a reduced level of both extracellular HBV-DNA and HBsAg was detected in the culture supernatant of mutant virus (Fig. 3D,C). Collectively, these results illustrated that Enh-II mutations contributed to attenuated HBV replication, reduced SHBs expression and alteration in relative levels of LHBs and SHBs, resulting in suboptimal virion and HBsAg secretion.

### Effect of Occult-associated mutations inside X-Promoter

**Figure 5:**
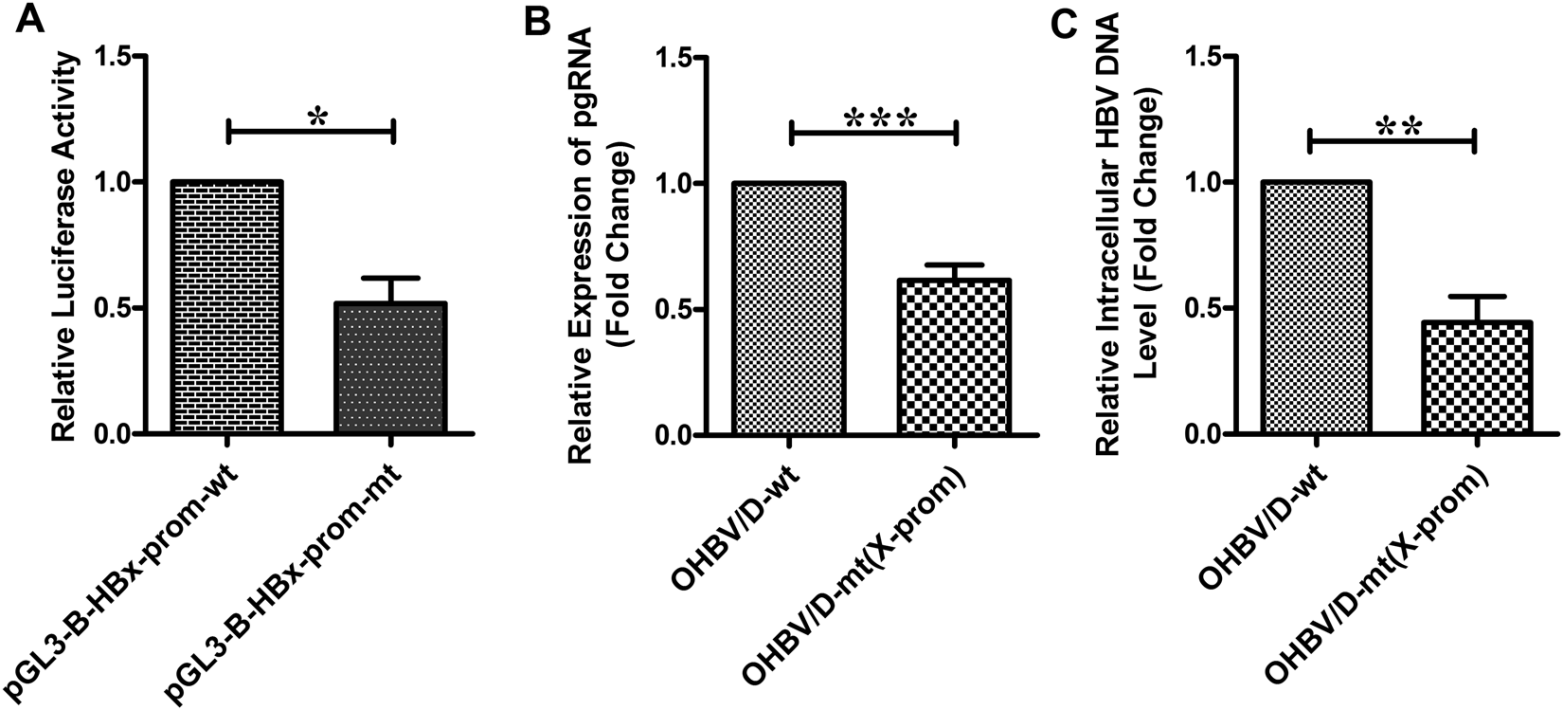
Effects of substitutions in X-promoter on promoter activity, pgRNA expression and HBV-DNA level. (**A**) Relative luciferase activities of the constructs pGL3-B-HBx-promoter-wt and pGL3-B-HBx-promoter-mt carrying wild-type and mutated X-promoter respectively cloned in pGL3-Basic vector. Relative expression of pgRNA (**B**) and intracellular HBV-DNA level (**C**) as measured by quantitative real-time PCR, following transfection of the full-length, wild-type HBV/D (HBV/D-wt) and corresponding X-promoter-mutated HBV [HBV/D-mt(X-prom)] in Huh7 cells. Paired t-test p values; *p < 0.05, **p < 0.005, ***p < 0.0005.

HBx is known to augment viral transcription as well as replication^16^. The expression of HBx is directed by the X-promoter, where four mutations were identified in OHBV/D. We first assessed the impact of these four mutations on the activity of the X promoter for which, the full-length promoter region of HBx with or without these mutations was cloned in pGL3 basic vector and the abilities of the constructs, pGL3-B-HBx-promoter-mt and pGL3-B-HBx-promoter-wt to drive the luciferase expression were studied following transfection in Huh7 cells. It was observed that the mutant X-promoter exhibited ~48% lower luciferase activity than the native one (Fig. 5A), implying a reduced intrinsic strength of the mutant X-promoter that in turn would also inhibit the expression of HBx. Further, transfection of full-length HBV bearing the four X-promoter mutations in Huh7 cells resulted in a significant decrease in both pgRNA (~1.6 fold) and intracellular HBV-DNA (~2.6 fold) relative to wild-type HBV (p < 0.005) (Fig. 5B,C), suggesting that these X-promoter mutations had the potential to negatively regulate HBV replication that may lead to the occult phenotype.

### Effect of mutations inside PreS2 and X protein as well as in Enh-II on generation of ER Stress

**Figure 6:**
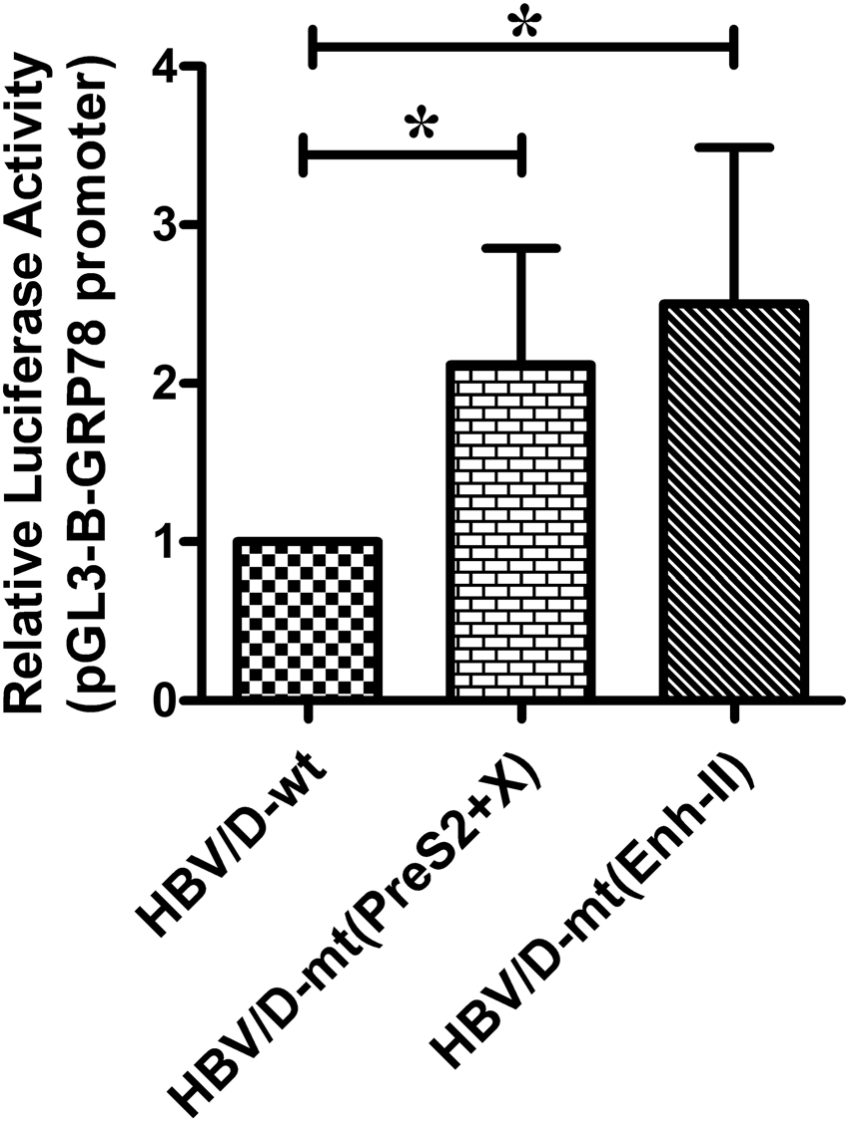
Effects of mutations in PreS2 and X-ORFs as well as in Enh-II on generation of ER stress by analyzing GRP78-promoter activity. The full-length, wild-type HBV (HBV/D-wt) along with the mutant variants harbouring mutations in (i) PreS2 and X-ORF [HBV/D-mt(PreS2 + HBX)] and (ii) Enh-II element [HBV/D-mt(Enh-II)] were co-transfected with pGL3-GRP78-promoter construct separately in Huh7 cells and the relative luciferase activities were determined after 48 hrs. Paired t-test p values; *p < 0.05.

We attempted to determine whether HBV harbouring the combined substitutions, P36L and I42L inside PreS2 and R26C and P33S in HBX could generate greater ER stress in infected hepatocytes than wild-type HBV. Since we observed altered expression ratio of LHBs to SHBs in HBV with mutated Enh-II, we also speculated that this mutant could result in up-regulation of the ER stress pathway in the host cells. Hence we studied the transcriptional activation of the promoter of GRP78, the pro-survival ER chaperon, cloned in pGL3-Basic vector in presence of these different HBV mutants. A significant increase in GRP78-promoter activity was detected when all these mutants were separately transfected in Huh7 cells, relative to wt-HBV (p < 0.03) (Fig. 6).

## Discussion

In the present study, we analyzed the prevalence of OBI in chronically HCV infected patients of Eastern India and performed an extensive molecular characterization of OHBV to gain an insight into plausible virological mechanisms that might be linked to the occult infection. While previous studies had illustrated the impact of HCV, particularly, the role of HCV core^17^ and NS2 protein^18^ in suppressing HBV replication and antigen expression and also highlighted the contribution of host immune response-related^3, 19^ and epigenetic mechanisms^20^ in induction of OBI, we have demonstrated that mutations in the coding and transcriptional regulatory regions of HBV could also play a pivotal role in the genesis of OBI. Thus our findings add to the existing knowledge on the mechanisms leading to the occult phenomenon.

OBI is relatively common in patients with chronic HCV infection and its prevalence had been reported to be 15–33% in different studies^3^. In India, the population prevalence of chronic HBV and HCV infection are ~2–4% and ~1% respectively^21^ but the sheer enormity of the population of this region accounts for a large chunk of the entire global pool of HBV and HCV carriers. However, till date only a single study reported the detection of OBI in 7.3% of chronic hepatitis C patients in southern India^22^. Here we noted that 17.8% of chronic HCV carriers of Eastern India carried OHBV. In contrast to previous reports suggesting a strong association between OBI and presence of anti-HBc and anti-HBs antibodies^2, 3^, we observed that the prevalence of OBI was particularly high (47.4%) in those HCV-infected patients without any evidence of prior HBV infection. A similar high incidence of OHBV had been described by Fukuda *et al*. who documented the presence of OBI in 50% of HCV infected patients without any serum markers of HBV infection^9^. Yotsuyanagi *et al*. also reported the detection of OHBV in sera of 53.8% HCC patients who were negative for HBsAg, anti-HBs, anti-HBc and anti-HCV^23^. It is unclear whether the complete seronegativity results due to gradual disappearance of all viral markers in the years after the resolution of an acute infection or whether it occurs from the beginning of the infection or whether both these two conditions (i.e presence and absence of serum HBV markers) may occur in various cases. Nonetheless, our findings reiterate the fact that sensitive HBV DNA amplification assays should be used for detection of OBI over HBV serological screening.

Owing to the very low titre of HBV-DNA in OBI, a very few studies had analyzed the full-length OHBV genome^24, 25^ while most had been restricted to analysis of one or two subgenomic regions^3^. Here we sequenced the full-length OHBV from 19 HCV patients, 17 of which belonged to genotype D. A robust genotype-matched comparison of OHBV/D sequences with HBV/D from HBsAg-positive chronic carriers in the same or different geographical settings led to the identification of mutations in PreS2, S, X, polymerase and in X-promoter and Enh-II of HBV that were potentially associated with occult status. We further studied the biological implications of these mutations in the generation of OBI.

Mutations within HBsAg, particularly in “a” determinant region, were one of the earliest known mechanisms leading to OBI^3^. We observed a striking preponderance of two “a” determinant mutations, T125M and P127T in OHBV as compared to the overt virus. These two mutations had been detected in OHBV from Greek^26^ and Egyptian blood donors^27^ while T125M was common in anti-HBc-positive blood donors of South-eastern India^28^. A decrease in the antigenicity of the HBsAg due to the presence of these mutations had been suggested previously^29^, although Zheng *et al*. reported that these substitutions were recognized by anti-HBs antibodies^30^. By *in vitro* experiments and *in silico* analysis, we demonstrated that these mutations did not alter the synthesis of HBsAg but resulted in changes in antigenicity, affecting its interaction with antibody and contributing to weak reactivity or negative results by diagnostic tests. The BeneSphera HBsAg Microwell ELISA, which employs specific anti-HBs monoclonal antibodies coating the solid phase to capture HBsAg, with polyclonal antibodies as the detection conjugate as well as the mouse monoclonal anti-HBs primary antibody used for Western blotting in this study were not efficient in detecting the S-mutants T125M and P127T. Thus our findings reinforced the need for extending the antibody repertoire of the current assays in order to detect these emerging HBsAg variants that are identified in occult HBV derived from HCV patients. It had been suggested that immunoassay that uses polyclonal antibodies for both capture and detection was associated with higher detection of mutants^31^ although this may also reduce assay sensitivity^30^ and specificity^32, 33^. Manufacturers could thus investigate the possibilities of using polyclonal capture antibody based assays, or mixtures of monoclonal antibodies that recognize both wild type and mutant HBsAg or tracer and capture antibodies against the pre-S region in order to circumvent the diagnostic failure related to S gene mutants of the “a” determinant. Further, these mutations in “a” determinant could accentuate the ability of the variant virus to evade the host immunity and persist in both unvaccinated and vaccinated hosts.

It is known that the replication of HBV-DNA proceeds through reverse transcription of 3.5-kb pgRNA, a reaction catalyzed by the viral polymerase^1^. Analysis of ORF-P revealed that 7 substitutions in rt and 2 in rh domains occurred at a significantly high frequency in OHBV than in virus from HBsAg-positive carriers. Our *in vitro* replication assay depicted a significant reduction in the replication efficiency of HBV in presence of these rt and rh mutations, which could partly explain the low copy numbers of HBV in OBI-positive patients. Further, the slow replication rates are often advantageous for viruses as by generating fewer virus-derived peptides, they could avoid being eliminated by host immune system and thereby have a greater likelihood of persistence in the host^34^.

HBV replication cycle is tightly coupled to viral gene expression that in turn is regulated by HBV promoters in conjunction with enhancers. The analysis of regulatory elements of OHBV revealed major changes in Enh-II that activates transcription from BCP and PreS2/S promoter. We noticed that the Enh-II mutations caused a marked reduction in pgRNA synthesis and a parallel decline in intracellular and extracellular HBV-DNA, an effect that is likely mediated through repression of core-promoter activity. Concurrently the inhibitory effect of these mutations on PreS2/S-promoter was reflected in the decrease in the levels of SHBs, low SHBs:LHBs ratio and diminished virion and HBsAg secretion. Thus it appears that these alterations in HBV life cycle evoked by Enh-II mutations may act conjointly to predispose the virus in acquiring the occult phenotype.

Further, four occult-associated mutations were discerned in X-promoter of HBV, of which the T1050G/A+ A1053G combination in HBV/D had been shown to be significantly associated with the development of HCC^35^. In a cohort of Chinese CHB patients, A1053G mutation was found to be associated with low HBV-DNA level, suggesting a negative impact of this mutation on HBV replication^36^. We observed that the transcription from the X-promoter was significantly weakened in presence of these mutations and coincided with a concomitant decline in HBV replication. Thus the X-promoter mutation, through possible modulation of X expression, could account for the low-level viremia seen in OBI-positive patients.

It is well established that chronic HBV infection is one of the major causes of advanced liver disease. PreS mutants as well as alteration in LHBs to SHBs ratio in the infected hepatocytes had been implicated in the induction of ER stress mainly from over-expressed or ER-retained LHBs in the face of lacking SHBs and thus contribute to disease progression^37^. In addition, the role of HBX in causing ER stress had also been documented^38^. Since OHBV isolates frequently carried mutations in PreS2, X and Enh-II, we envisaged that all these mutations could induce greater ER stress signals in the host cell than wt-HBV. Commensurate with this hypothesis, we showed that the activation of GRP78-promoter was much stronger for PreS2-X or Enh-II mutant than wild-type, suggesting increased potential of OHBV to trigger ER stress, which could sensitize the hepatocytes to injury and favour the advancement of liver disease.

Hence, by analyzing the full-length genome of OHBV from HCV carriers, this study has for the first time identified multiple mutations in HBV that could potentially lead to the induction and maintenance of HBV infection in an occult status. Phenotypic analysis of HBV carrying these specific mutations in Enh-II and S-ORF revealed that these mutations were associated with reduced synthesis and secretion of HBsAg as well as its impaired detection by immunoassays, which in turn can lead to HBsAg negativity in OHBV infected patients. Additionally, mutations inside ORF-P, X promoter and Enh-II region were significantly correlated with attenuated virus replication and could account for the low level of HBV DNA in the serum of OBI positive patients. We have shown the functional consequences of the mutations individually but since most of the mutations co-exist in OHBV, it is likely that they act “synergistically” and have a significant cumulative impact in escorting the virus towards its occult phenotype. Our results also extended the functional ambit of OHBV to include their enhanced propensity to generate ER stress and consequently liver damage. Given that the reactivation of HBV after HCV treatment with direct-acting antivirals is an emerging issue in coinfected patients, it is becoming increasingly important to detect and monitor patients with OBI and to have a clearer understanding of the biology of the occult virus so as to reduce the risk of end-stage liver disease in future.

## Materials and Methods

### Patients

Treatment-naive patients with chronic HCV infection, having serum HCV-antibody positivity (>6 months) but negative for HBsAg were included in the study from the Hepatology Clinic of School of Digestive and Liver Diseases, Institute of Post Graduate Medical Education and Research (I.P.G.M.E.&R.), Kolkata, India. Patients with HIV infection or other recognized causes of chronic liver disease or any systemic illness were excluded. Five millilitre blood was collected from all patients, the sera were separated and stored at −80 °C until use. Written informed consents were obtained from all patients and the access to human samples and all experimental protocols were carried out in accordance with the approved guidelines of the Ethical Review Committee of I.P.G.M.E.&R.

### Testing of Liver Enzymes and Serological Markers of HBV

The levels of liver enzymes alanine aminotransferase (ALT; normal range 2–40 IU/litre) and aspartate aminotransferase (AST; normal range 2–45 IU/litre) in each serum sample were analyzed using commercially available kits (Bayer Diagnostics). The tests for serum HBsAg, anti-HBc and anti-HBs were accomplished using BeneSphera HBsAg Microwell ELISA kit (Avantor Performance Materials), DS-EIA-ANTI-HBsAg ELISA kit (DSI S.r.l.) and DS-EIA-ANTI-HBc ELISA kit (DSI S.r.l.) respectively, according to the manufacturers’ protocols. The analytical sensitivity of BeneSphera HBsAg Microwell ELISA kit was 0.2 IU/ml, whereas the diagnostic sensitivity and specificity of this kit was 100% and 99.5% respectively.

### Screening for OBI

Viral DNA was extracted from 1 ml sera samples with QIAamp DNA Blood Midi kit in accordance with the manufacturer’s instructions. (Qiagen Inc.) DNA was eluted in 200 μl of sterile distilled water that was concentrated to 30 μl using a DNA concentrator (Savant DNA Speedvac, Thermo Scientific). This concentrated DNA extract was analyzed for OHBV by performing 3 different nested PCR assays using two sets of forward and reverse primers specific for each of surface, core and Polymerase/X genes respectively (Supplementary Table S1). For the first amplification, the 20-μl reaction mixture contained 5 μl of extracted/concentrated DNA, 0.2 μM concentrations for each forward and reverse primer, 200 μM dNTPs, 2.0 U of ExPrime Taq DNA polymerase (Genet Bio Inc.) and 1X reaction buffer. The mixtures were amplified with an initial denaturation at 94 °C for 5 min followed by 45 cycles at 94 °C for 30 s, 56 °C for 60 s, and 72 °C for 2 min. There was a final extension at 72 °C for 7 min. For the second round PCR, 5 μl of product from the above-described reaction mixture was used with second set of forward and reverse primers and amplified under the same conditions. Appropriate no-template controls (water), negative controls (serum DNA extracts from HBV/HIV/HCV-seronegative healthy subjects) and positive controls (serum DNA extracts from HBsAg-positive subjects and/or cloned plasmid containing HBV genome) were included during each amplification step. Only cases that showed positivity in at least 2 viral sub-genomic regions were considered to be OHBV-infected.

### Quantification of OHBV-DNA

OHBV-DNA was quantified by real time PCR using Robogene HBV Real-time Quantification Kit (Germany) following the protocol recommended by the manufacturer and the conversion between IU/ml and copies/ml is based on the conversion factor of 5.0, indicating that 5 HBV copies/ml measured by this assay is equivalent to 1 IU/ml.

### Amplification and sequencing of complete OHBV Genome

From OBI-positive cases, OHBV-DNA was amplified by a nested PCR approach to obtain overlapping sub-genomic fragments, covering the entire viral genome. The first PCR was carried out in 25 μl reaction mixture containing 10 μl of extracted/concentrated viral DNA, 0.2 μM of each of the primers HBVP1 and HBVP2^39^ (Supplementary Table S2), 5 U of High Fidelity PCR Enzyme mix (Thermo Scientific/Fermentas), 250 μM dNTPs and 1X High Fidelity PCR buffer with 1.5 mM MgCl_2_. The optimized cycling protocol included 5 min of incubation at 94 °C, followed by 45 cycles each of 94 °C for 40 sec, 60 °C for 1 min, and 68 °C for 3 min 10 sec with 5 sec increment/cycle and a final extension at 68 °C for 7 min. For the second-round PCR, 5 μl of the first PCR product was used as template along with primer pairs MP1-R5 and F3-MP2 or F3-R10, C1861F-R4, F7-R2 and F1-SP2 (Supplementary Table S2) and all other reagents were added in same concentrations as given above. The cycling conditions were usually 45 cycles at 94 °C for 40 sec, 56 °C for 30 sec, and 68 °C for a time dependent on the expected product size (1 min per kb). The PCR products were purified by QIAquick PCR purification kit (Qiagen) and sequenced directly using BigDye terminator v3.1 cycle sequencing kit (Applied Biosystems) on an automated DNA sequencer. Sequence editing and analysis were performed using Seqscape V2.5 software.

### Sequence Analysis

To determine the genetic affiliation of OHBV strains, the full-length sequences obtained in the study were compared with representative sequences of ten HBV genotypes (A–J) retrieved from GenBank. Alignments were carried out using CLUSTALX software and a phylogenetic tree was constructed by the neighbour joining (NJ) method using the Jukes–Cantor model in MEGA software version 6^34^. To identify the potential mutations in HBV that are associated with occult status, the complete sequences of OHBV belonging to genotype D (OHBV/D) were compared with 167 HBV/D sequences derived from HBsAg-positive carriers that comprised of 62 sequences from Eastern India (EI) and 105 sequences from other geographical locations (WRLD), all of which are available in GenBank. The candidate mutations were identified by conducting multiple sequence alignment by CLUSTALX as implemented in MEGA6.

### Antigenicity Plot

Antigenicity profile of selected epitope region of HBsAg [aa. 124–147] with or without the occult-associated substitutions was investigated by Kolaskar and Tongaonkar Antigenicity Prediction method^40^ using Immune Epitope Database Analysis Resource (http://tools.immuneepitope.org/bcell/) and applying a window size: 5 and threshold: 1.

### Cloning of full-length HBV and introduction of Occult-associated mutations

Full-length HBV genome of genotype D isolated from archival serum sample of a HBsAg- and HBeAg-positive chronic Hepatitis B (CHB) patient was amplified using primers HBVP1 and HBVP2, cloned into pJET1.2 vector using CloneJET PCR Cloning kit (Fermentas) and used as wild-type (wt) HBV. All OHBV-specific mutations, identified in this study, except those in ORF-P, were introduced separately in wt-HBV clone (pJET-HBV-wt) by Site-Directed Mutagenesis (Agilent Technologies) with specific mutagenic oligonucleotides (Supplementary Table S3) and confirmed by sequencing. The substitutions detected in reverse transcriptase (rt) and RNaseH (rh) domains of ORF-P of OHBV were introduced in replication competent HBV/D cloned in pTriEX-Mod vector (pTriEX-Mod-HBV-D) (a kind gift from Prof. Fabien Zoulim, INSERM, France) by cassette insertion method^34^. Briefly, an 1802 bp region of ORF-P (nt.3164–1783) was amplified using primers F4 and R10 (Supplementary Table S2) from pJET-HBV-wt and from previously sequenced OHBV/D DNA included in the study. The 1802 bp amplicons were digested using *AvrII* and *NcoI* restriction enzymes (Fermentas) and individually used to replace the corresponding region of HBV/D in pTriEX-Mod-HBV-D. Thus, two constructs, pTriEXMod/HBV-D-wt (having rt and rh regions of wt-HBV) and pTriEX-Mod/HBV-D-mt (with rt and rh region of OHBV) were generated that were isogenic, except for their rt and rh domains.

### Cell Culture and Transfection

To determine the effect of occult-associated mutations inside the ORF-S, ORF-X, or regulatory regions of HBV on its own life cycle or on the host cells, as compared to the wt-HBV genome, full-length wt or different mutant (mt)-HBV genomes were released from pJET1.2 vector by *SapI* digestion and these linear full-length HBV monomers were transfected in Huh7 cells^41^. To evaluate the effects of mutations inside rt and rh regions of ORF-P, replication competent pTriEXMod/HBVD-WT and pTriEX-Mod/HBV-D-MT plasmids were used directly for transfection assay. Huh7 cells were seeded into 6 well plates at a concentration of 2 × 10^5^ cells/well in DMEM (HIMEDIA) with 10% FBS (Gibco). Sixteen hours post seeding, cells were transiently transfected separately with 2 µg of purified Sap*I* digested, full-length linear monomeric HBV (wt and appropriate mt genome) or pTriEXMod/HBV-D plasmids (wt/mt) along with pRL-CMV *Renilla Luciferase* Reporter Vector (Promega), which served as transfection normalization control, using Lipofectamine 2000 transfection reagent (Invitrogen).

### Viral DNA, RNA and protein analyses

Forty-eight hours post-transfection, Huh7 cells were harvested and HBV-DNA was isolated from both intracellular core particles^34^ as well as from culture supernatant. To isolate the intracellular HBV DNA from the viral core particles, HBV transfected Huh7 cells were lysed with lysis buffer (50 mM Tris-HCL, pH = 8; 1 mM EDTA; 1% NP40), centrifuged at a speed of 10,000 rpm for 1 minute at room temperature followed by DNase I (Roche) and RNase A (Fermentas) digestion at 37 °C for 3 hours. After that the HBV core particles were digested with Proteinase K (Roche) overnight at 42 °C. Finally the intracellular viral DNA was extracted with phenol, chloroform, isoamyl alcohol (25:24:1) and precipitated with 3 M NaOAc (pH = 5.2) and ethanol. Extracted viral DNA was quantified by Real Time PCR with SYBR Green Master mix (Applied Biosystems) as described by Garson *et al*.^42^. A 40 cycle real-time PCR was performed in QuantStudio 7 Flex (Applied Biosystems) using primer-pair F4-R3 (Supplementary Table S2) with the following thermal condition: 94 °C for 30 sec, 56 °C for 15 sec, 72 °C for 30 sec. A 5 point standard curve was prepared by the serial dilution of the WHO International Standard for HBV DNA NAT assays, 97/746 (5 × 10^5^ IU per vial). HBV-DNA was also isolated from cell culture supernatant with QIAamp Blood Kit (Qiagen Inc.) and quantified similarly.

Total RNA was extracted from transfected cells and cDNA was generated using High-Capacity cDNA Reverse Transcription kit (Applied Biosystems) and the expression of pregenomic RNA (pgRNA), PreS1 and PreS2 transcripts were determined by Real-Time PCR using specific primers (Supplementary Table S2) and SYBR Green Master mix. The cycling conditions consisted of an initial step at 95 °C for 10 min followed by 40 cycles of 95 °C for 25 sec, 56 °C for 25 sec and 72 °C for 25 sec (with fluorescence acquisition). Amplification was followed by a melting curve analysis over a linear temperature increase (0.05 °C per sec) from 60 °C to 95 °C. All assays were performed in triplicates and the expression of *Renilla luciferase* was used to normalize the transfection efficiency. Additionally, the levels of intracellular HBV envelope proteins were determined by immunoblotting with mouse monoclonal anti-HBs primary antibody (anti-Ad/Ay; MyBioSource) while the extracellular HBsAg in culture supernatant was determined by ELISA using BeneSphera HBsAg Microwell ELISA kit.

### Dual luciferase reporter gene assay

The full-length, wild-type and mutant HBx-promoter and Enh-II regions were separately amplified from pJET-HBV-wt and from previously sequenced OHBV-DNA, using specific primers (Supplementary Table S2). The promoter-amplicons were inserted into the *KpnI*/*XhoI* sites of pGL3-Basic vector (Promega) to generate pGL3-B-HBx-promoter-wt and pGL3-B-HBx-promoter-mt constructs. The enhancer-II-amplicons were inserted at similar sites in pGL3-Promoter vector (Promega) to generate pGL3-P-wt-Enhancer-II and pGL3-P-mt-Enhancer-II. All constructs were verified by sequencing and co-transfected with pRL-CMV vector in Huh7 cells. In addition, the full-length promoter of GRP78 cloned in pGL3-Basic vector^43^ was transfected in Huh7 cells along with pRL-CMV vector and linear monomers wild-type or mutant-HBV (carrying Enh-II mutations as well as the combined PreS2 and X mutations). In all cases, after forty eight hours *Firefly* and *Renilla luciferase* activities were measured using Dual-Luciferase Reporter Assay kit (Promega) in luminometer.

### Statistical analysis

Data were expressed as median (range) or mean ± standard deviation as appropriate. Statistical analysis was performed using GraphPad Prism 5.0. Fisher’s exact test was performed to make pairwise group comparisons of mutation frequencies. For all tests, p values < 0.05 was considered significant.

### Nucleotide sequence accession numbers

The complete nucleotide sequences of 17 OHBV/D isolates are available in GenBank under accession numbers KU668433-KU668449.

## Electronic supplementary material

Supplementary Information
