# Supplementary material for: Synergistic impact of mutations in Hepatitis B Virus genome contribute to its occult phenotype in chronic Hepatitis C Virus carriers
Source: Sci Rep. 2017 Aug 29;7:9653. doi: 10.1038/s41598-017-09965-w (PMC5574988; doi:10.1038/s41598-017-09965-w)
Supplement: Supplementary file 1 — Supplementary Information [file 41598_2017_9965_MOESM1_ESM.doc]

**Synergistic impact of mutations in Hepatitis B Virus genome contribute to its occult phenotype in chronic Hepatitis C Virus carriers**

Rajiv Kumar Mondal^1^, Mousumi Khatun^1^, Priyanka Banerjee^1^, Alip Ghosh^1^, Sumanta Sarkar^1^, Amal Santra^1^, Kausik Das^2^, Abhijit Chowdhury^2^, Soma Banerjee^1^ and Simanti Datta^1^*

^1^Centre for Liver Research, School of Digestive and Liver Diseases, Institute of Post Graduate Medical Education and Research, Kolkata, INDIA

^2^Department of Hepatology, School of Digestive and Liver Diseases, Institute of Post Graduate Medical Education and Research, Kolkata, INDIA

***Corresponding Author:** Dr. Simanti Datta

Centre for Liver Research,

School of Digestive and Liver Diseases,

Institute of Post Graduate Medical Education and Research,

244, A.J. C. Bose Road,

Kolkata-700020, INDIA.

Tel- (91)-(033)-2223 5435; Fax- (91)-(033)-2223 6383

E-mail- seemdatt@gmail.com

**Supplementary Table S1: List of Primers used for screening of OHBV-DNA**

| **Region of HBV** | **Primers for PCR** | | **Primer Sequence** | **Amplicons (bps)** |
| --- | --- | --- | --- | --- |
| **Surface** | **1^st^ round of PCR** | **F8** (Sense) | 5′-ATTTGCATACTCTTTGGAAGGC-3′ | 1733 |
|  |  | **R9** (Antisense) | 5′-TAGGAGTTCCGCAGTATGGA-3′ |  |
|  | **2^nd^ round of PCR** | **F3** (Sense) | 5′-CGCCTCATTTTGTGGGTCAC- 3′ | 1389 |
|  |  | **R8** (Antisense) | 5′-CTTTGACAAACTTTCCAATCAAT-3′ |  |
| **Core** | **1^st^ round of PCR** | **CF** (Sense) | 5′-ACTGTTCAAGCCTCCAAGCT-3′ | 1814 |
|  |  | **R4** (Antisense) | 5′-AGAGGACAAACGGGCAACA-3′ |  |
|  | **2^nd^ round of PCR** | **HBCF** (Sense) | 5′-GACAAGCTTGGGACATTGACCC-3′ | 1377 |
|  |  | **R3** (Antisense) | 5′-AACTGGAGCCACCAGCAG-3′ |  |
| **Polymerase/X** | **1^st^ round of PCR** | **F5** (Sense) | 5′-GATGTGTCTGCGGCGTTTTA-3′ | 1405 |
|  |  | **R10** (Antisense) | 5′-CAGCCTCCTAGTACAAAGAC-3′ |  |
|  | **2^nd^ round of PCR** | **F9a** (Sense) | 5′-TACCACAAGAGCACATTATACA-3′ | 939 |
|  |  | **R11** (Antisense) | 5′-CTTTAACCTAATCTCCTCCC-3′ |  |

**Supplementary Table S2: List of Primers used for amplification and sequencing of HBV gen**ome, viral load determination and quantification of gene expression

| **Primer Name** | **Primer Sequences** | **Location (nt)*** |
| --- | --- | --- |
| **F4^a,c^** (Sense) | 5′-CTCAGGCCATGCAGTGGAA-3′ | 3164-3182 |
| **R3^a,c^** (Antisense) | 5′-AACTGGAGCCACCAGCAG-3′ | 57-74 |
| **HBVP1^b,#^** (Sense) | 5′-CCGGAAAGCTTGAGCTCTTCTTTTTCACCTCTGCCTAATCA-3′ | 1821-1841 |
| **HBVP2^b,#^** (Antisense) | 5′-CCGGAAAGCTTGAGCTCTTCAAAAAGTTGCATGGTGCTGG-3′ | 1806-1825 |
| **MP1^b,$^** (Sense) | 5′-GAGCTCTTCTTTTTCACCTCTGCCTAATCA-3′ | 1821-1841 |
| **R5^b,c^,**^$^ (Antisense) | 5′-AAAGCCCAAAAGACCCACAAT-3′ | 997-1017 |
| **F3^b,c^,**^$^ (Sense) | 5′-CGCCTCATTTTGTGGGTCAC-3′ | 2801-2820 |
| **MP2^b,$^** (Antisense) | 5′-GAGCTCTTCAAAAAGTTGCATGGTGCTGG-3′ | 1806-1825 |
| **R10^b,c^** (Antisense) | 5′-CAGCCTCCTAGTACAAAGAC-3′ | 1764-1783 |
| **C1859F^b,c^** (Sense) | 5′-ACTGTTCAAGCCTCCAAGCT-3′ | 1859-1878 |
| **R4^b,c,f^** (Antisense) | 5′-AGAGGACAAACGGGCAACA-3′ | 462-480 |
| **F7^b,c^** (Sense) | 5′-TGTGCACTTCGCTTCACCTC-3′ | 1578-1597 |
| **R2^b,c^** (Antisense) | 5′-AAATTACCACCCACCCAGG-3′ | 2109-2127 |
| **F1^b,c^** (Sense) | 5′-CACAAGAGGACTCTTGGACT-3′ | 1653-1672 |
| **SP2^b,c^** (Antisense) | 5′-GTATGGTGAGGTGAACAATG-3′ | 2039-2058 |
| **F5^c,f^** (Sense) | 5′-GATGTGTCTGCGGCGTTTTA-3′ | 376-395 |
| **F10^c^** (Sense) | 5′-GACCACCAAATGCCCCTATC-3′ | 2298-2317 |
| **PreG_F^d^** (Sense) | 5′- CACCTCTGCCTAATCATC-3′ | 1826-1843 |
| **PreG_R^d^** (Sense) | 5′- GGAAAGAAGTCAGAAGGCAA-3′ | 1955-1974 |
| **PreS1(N)_F^e^** (Sense) | 5′-TTCCACCAGCAATCCTCTGG-3′ | 2862-2881 |
| **2956_R^e^** (Antisense) | 5′- TGGGATTGAAGTCCCAATCTGG-3′ | 2935-2956 |
| **Xprom_F^g^** (Sense) | 5′-TACTGGTACCTCCTGTTAACAG-3′ | 957-968 |
| **Xprom-wt_R^g^** (Antisense) **(Sense)** | 5′-TACACTCGAGGGGAGAGAACAAC-3′ | 1342-1354 |
| **Xprom-mt_R^g^** (Antisense) | 5′-TACACTCGAGGGGATAGAACAAC-3′ | 1342-1354 |
| **EnhII-wt_F^h^** (Sense) | 5′-TACTGGTACCTCTTGCCCAAGGTC-3′ | 1636-1649 |
| **EnhII-mt_F^h^** (Sense) | 5′-TACTGGTACCTATTGCCCAAGGTC-3′ | 1636-1649 |
| **EnhII_R^h^** (Antisense) | 5′-TACACTCGAGAACTCCTCCCAGTC-3′ | 1728-1741 |
| **18sF^i^** (Sense) | 5′-GTAACCCGTTGAACCCCATT-3′ | - |
| **18sR^i^** (Antisense) | 5′-CCATCCAATCGGTAGTAGCG-3′ | - |
| **Rluc_F^j^** (Sense) | 5′-GGAATTATAATGCTTATCTACGTGC-3′ | - |
| **Rluc_R^j^** (Antisense) | 5′-CTTGCGAAAAATGAAGACCTTTTAC-3′ | - |

* = nt. positions are given according to HBV sequence with accession no. AF121242 obtained from GenBank. ^a^Primers used for HBV-DNA quantification by Real-Time PCR, ^b^Primers used for PCR amplification, ^c^Primers used for sequencing, ^d^Primers used for pregenomic RNA quantification by Real-Time PCR, ^e^Primers used for PreS1 mRNA quantification by Real-Time PCR, ^f^Primers used for PreS2 mRNA quantification by Real-Time PCR, ^g^Primers used for cloning of HBx-promoter into pGL3-Basic vector, ^h^Primers used for cloning of Enhancer-II region into pGL3-Promoter vector,

^i^Primers used for 18S rRNA expression, ^j^Primers used for *Renilla luciferase* expression.

^#^Günther S, et al. J Virol 1995;69:5437-5444, ^$^Pollicino T, et al. Hepatology 2007;45:277-285.

**Supplementary Table S**3: List of Primers used for Site Directed Mutagenesis in this study

| **Primer Name** | **Primer Sequences** | **aa/nt changes introduced** |
| --- | --- | --- |
| **Small(125/127)_SDM_F** | 5'-CCATGCCGGACCTGCACGACTCCTGCTCAAGGAACC-3' | T125M and P127T inside ORF-S |
| **Small(125/127)_SDM_R** | 5'-GGTTCCTTGAGCAGGAGTCGTGCAGGTCCGGCATGG-3' |
| **C1637A_F** | 5'-CCGTGAACGCCCACCACTTATTGCCCAAGGTCTTAT  ATAAG-3' | C1637A inside Enhancer II |
| **C1637A_R** | 5'-CTTATATAAGACCTTGGGCAATAAGTGGTGGGCGT  TCACGG-3' |
| **T1676A_F** | 5'-GAGGACTCTTGGACTCTCAGTAATGTCAACGACC  GAC-3' | T1676A inside Enhancer II |
| **T1676A_R** | 5'-GTCGGTCGTTGACATTACTGAGAGTCCAAGAG  TCCTC-3' |
| **Xp1050G/53G/59T_F** | 5'-GTGGTTATCCTGCGTTGATGCCTTTGTATGCCT  GTATTC-3' | T1050G, A1053G and C1059T inside X promoter |
| **Xp1050G/53G/59T_R** | 5'-GAATACAGGCATACAAAGGCATCAACGCAGGA  TAACCAC-3' |
| **XpC1350A_F** | 5'-GATAACTCTGTTGTTCTATCCCGCAAATATACATC-3' | C1350A inside X promoter |
| **XpC1350A_R** | 5'-GATGTATATTTGCGGGATAGAACAACAGAGTTATC-3' |
| **PreS2(36/42)_F** | 5'-CAGTAAACCCTGTTCTGACTACTGCCTCTCCCT  TATCGTCAATCTTCTC-3' | P36L and I42L inside ORF-PreS2 |
| **PreS2(36/42)_R** | 5'-GAGAAGATTGACGATAAGGGAGAGGCAGTA  GTCAGAACAGGGTTTACTG-3' |
| **X(26/33)_F** | 5'-GCGCTGAATCCTGCGGACGACCCTTCTCGGGG  TCGCTTGGGTCTC-3' | R26C and P33S inside ORF-X |
| **X(26/33)_R** | 5'-GAGACCCAAGCGACCCCGAGAAGGGTCGTCC  GCAGGATTCAGCGC-3' |

* = nt. positions are given according to HBV sequence with accession no. AF121242 obtained from GenBank

**Supplementary Figure S1.**

**Supplementary Figure S1:** **Phylogenetic tree analysis of full-length sequences of 19 OHBV isolates from HCV patients** along with 33 reference sequences of HBV belonging to different genotypes (A–J) derived from GenBank, including 6 sequences of HBV from non-human primates. HBV sequences from GenBank are indicated by their genotypes, accession numbers and country of origin. The OHBV sequences from HCV patients (OHBV/HCV) determined in the study are indicated by the symbol ♦. The phylogenetic tree was built using the Jukes–Cantor model and neighbour joining method by MEGA6 and bootstrap resampling and reconstruction were carried out 5000 times.

**Supplementary Figure S2.**

**A)**

**
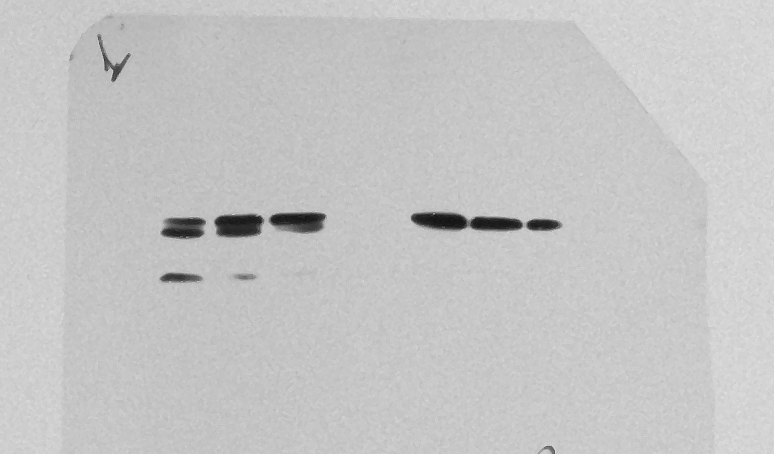
**

**Anti-HBsAg**

**HBV/D-wt**

**other**

**HBV/D-mt (HBsAg)**

**Others**

**
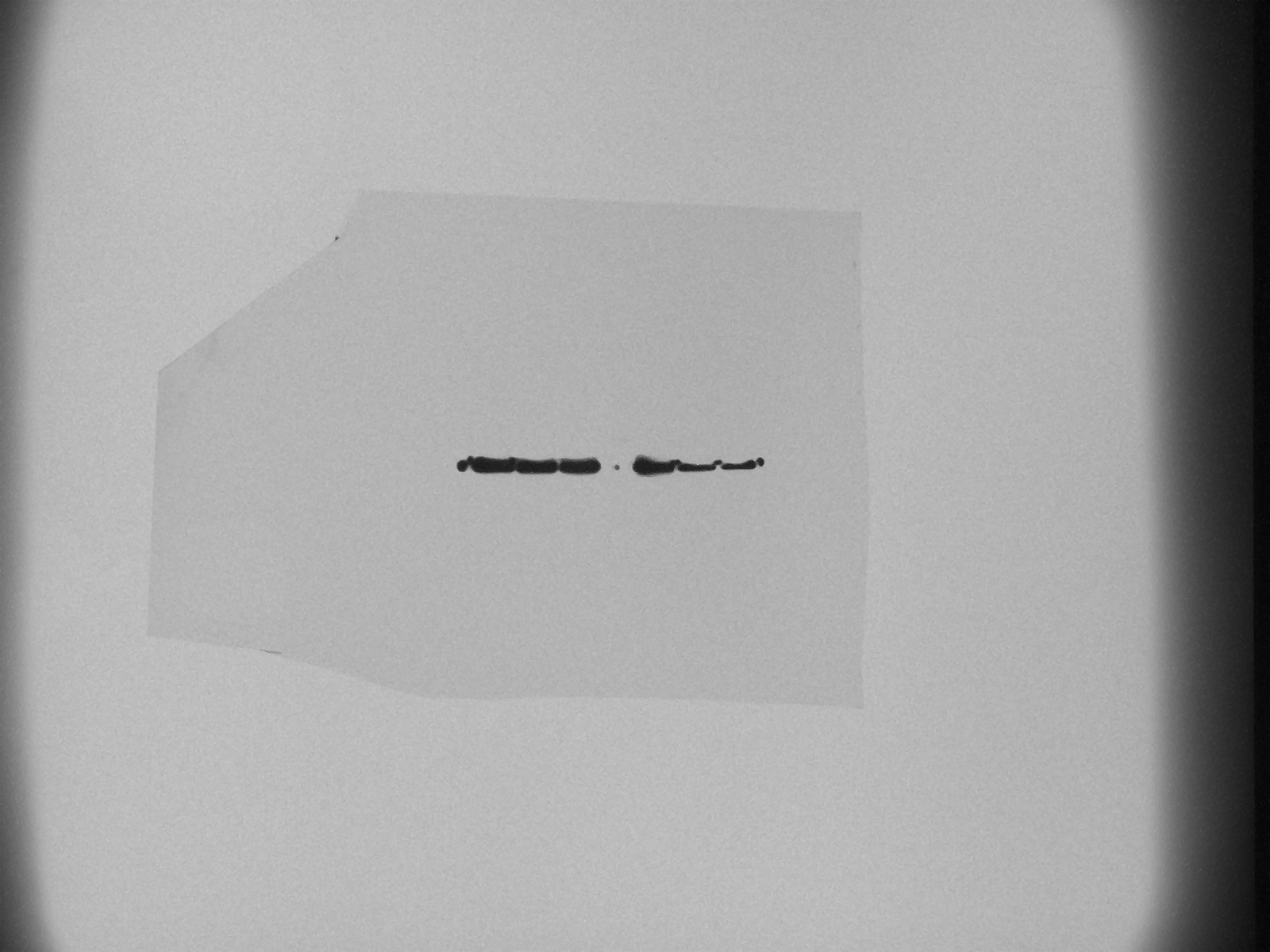
**

**Anti-α-Tubulin**

**HBV/D-wt**

**other**

**HBV/D-mt (HBsAg)**

**Others**

**Anti-α-Tubulin**

**B)**

**Others**

**HBV/D-wt**

**HBV/D-mt (Enh-II)**


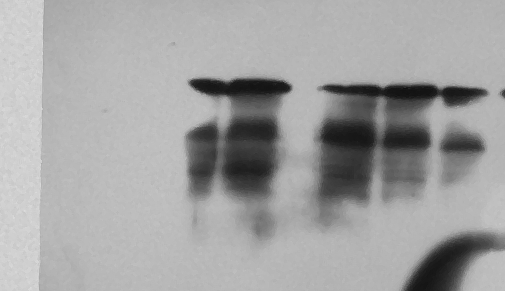


**Other**

**Anti-HBsAg**


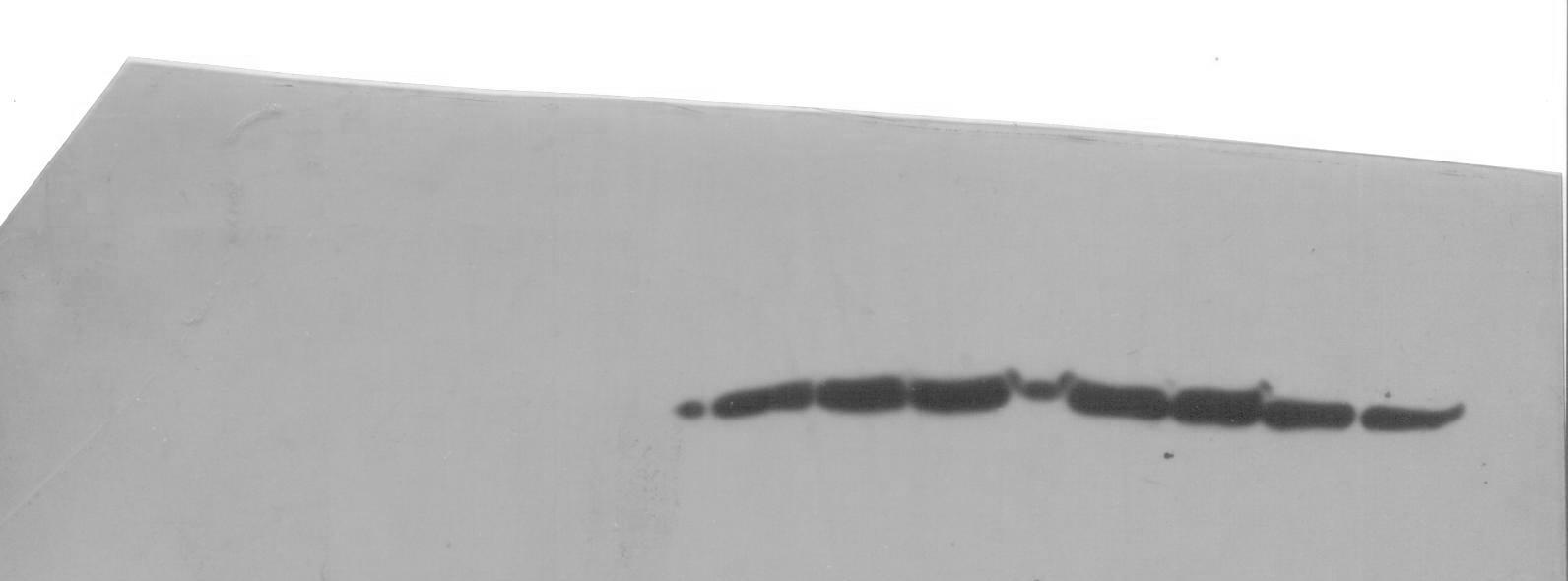


**Others**

**HBV/D-wt**

**HBV/D-mt (Enh-II)**

**Others**

**Supplementary Figure S2.** The full-length blots for **(A)** detection of HBsAg following transfection of wild-type HBV/D (HBV/D-wt) and mutant construct [HBV/D-mt(HBsAg)] carrying S- substitutions, T125M and P127T in Huh7 cells and **(B)** expression of HBV envelope proteins following transfection of wild-type HBV/D (HBV/D-wt) and corresponding Enh-II-mutated HBV [HBV/D-mt(Enh-II)] in Huh7 cells. The SDS-PAGE gel electrophoresis and probing with monoclonal anti-HBs primary antibody or anti-α tubulin antibody was carried out to detect envelope proteins of HBV and α-Tubulin (that served as loading control). After the proteins were electrotransferred to a PVDF membrane, the membranes were cut around the ~45 kDa protein marker band. The upper parts of the membranes were used for detection of α-Tubulin (~52 kDa) and the lower parts were used for detection of HBV envelope protein(s) (24 kDa - 42 kDa). The lanes with wild-type and mutant constructs used in this paper are indicated while other unrelated mutants are not disclosed.
